# Opportunistic pathogens and polycocktail drugs fuel dynamic public health threats during the opioid crisis

**DOI:** 10.1371/journal.pone.0326200

**Published:** 2025-08-12

**Authors:** John H. Boman, Nara Souza, Jasmine Light, Dawson Holt, Shannon Jones, Angelic King, Mahjida Berryman, Sarah A. Shuda, Mia Borrelli, Barry K. Logan, Amanda L. A. Mohr, Hans Wildschutte

**Affiliations:** 1 American University, Justice, Law & Criminology, School of Public Affairs, Washington, D.C., United States of America; 2 Department of Biological Sciences, Bowling Green State University, Bowling Green, Ohio, United States of America; 3 Toledo-Lucas County Health Department, Toledo, Ohio, United States of America; 4 Center for Forensic Science Research and Education, Fredric Rieders Family Foundation, Horsham, Pennsylvania, United States of America; Universidade dos Açores Departamento de Biologia: Universidade dos Acores Departamento de Biologia, PORTUGAL

## Abstract

More than 100,000 preventable deaths have occurred each year in the United States since 2021 from intravenous drug use. A challenging problem for clinicians, researchers, and public health workers involves understanding a dynamic opioid crisis for implementing state and national policy to support the rehabilitation and treatment of individuals who inject drugs. To address these issues, this study used drug needles obtained from people who inject drugs to address three goals: First, based on a forensic analysis, what narcotics are contained in the needles? Second, are there non-viral pathogens contained in the needles? Third, if pathogens are identified, can potential infections be treated with common medication? Results demonstrate that 27 total psychoactive substances (most listed in the Drug Enforcement Administration’s schedule of controlled substances) were present in 50 randomly selected syringes. The average used drug needle contained eight psychoactive substances. In addition to drugs, non-viral culturable microbes in used syringes were identified. The most problematic of these was an opportunistic fungal pathogen, *Candida parapsilosis*, that persists on skin and for which few medical options exist for treatment. To thus facilitate antifungal discovery, soil-derived bacteria that inhibit the growth of *Candida* pathogens were identified. Transposon mutagenesis was utilized to discover the biosynthetic gene cluster involved in antifungal activity which encoded a non-ribosomal peptide synthetase. Although limited to syringes from only one mid-western city, results suggest that potentially fatal, emergent, and opportunistic pathogens may have the ability to persist in used drug syringes. While this finding may be alarming to public health and public safety officials, the identification of bacteria which inhibits the growth of *Candida parapsilosis* poses a foundation for antifungal drug discovery.

## Introduction

Largely driven by opioids like fentanyl, the drug overdose crisis has triggered a grim public health event in the United States (U.S.). With over 100,000 overdose fatalities each year since 2021 [1], opioid overdoses have now surpassed car crashes, accidental falls, and suicide to become the leading cause of preventable and accidental death in the U.S. [2,3]. Due to its severity across the nation, it is critical that researchers, public health officials, and criminal justice practitioners collaborate to understand the dynamic nature of the crisis in order to determine the most effective policies and treatments to reduce overdoses and deaths [4,5].

Among people who inject drugs (PWID), needle sharing is common even though national efforts have been in place to eliminate the practice including education centers and harm reduction programs [6,7]. Thus, understanding the types of drugs and microbial contaminants that are injected and may be passed among PWID is of highest concern. Although opioid use is common practice among PWID, knowing the presence of all injected substances is necessary to comprehend an ongoing crisis. Regarding infections, viruses such as HIV and Hepatitis can be easily spread via intravenous needle use [8], yet represent only a portion of microbes that can cause serious and life-threatening medical conditions. Opportunistic pathogens usually do not cause disease in healthy individuals, but PWID are an immunocompromised population due to poor hygiene and irregular sleep patterns, two factors which contribute to a high risk of infection. Since intravenous drug use provides the chance for microbes to be delivered directly to the bloodstream, the potential for opportunistic pathogens to infect PWID is a real threat.

If PWID were exposed to a variety of drugs and pathogens, they may be best detected in a ‘hot spot’ for opioid use. Ohio is consistently one of the most disproportionately affected areas for illicit drug use and overdoses in the U.S. Since 2014, it has ranked in the top five states for the total number of fatal overdoses and top ten states in fatal overdose rate [9]. Lucas County, Ohio – with a population of 431,279 [10] – is located in the northwest region and home to the mid-major city of Toledo; the area has ranked in the top 10% of Ohio counties for overdose volume for the past decade [11]. Designated as a qualified opportunity zone by the U.S. federal government [12], Toledo has struggled to recover from years of structural, financial, and racial inequality. As of 2023, the greater Toledo area is among the highest region in Ohio in terms of crime [13], unemployment [14], welfare [14], socioeconomic challenges [15], and drug use [12].

In response to the drug crisis, the Toledo-Lucas County Health Department (TLCHD) attempts to reduce overdoses and infections through a variety of harm reduction practices such as targeted naloxone distribution, overdose surveillance, and the Northwest Ohio Syringe Service program (NOSS) which provides clean needles to PWID to prevent the spread of viral disease. In 2023, the NOSS distributed a total of 284,123 clean needles to the community [16]. In collaboration with the TLCHD’s NOSS program, we sought to identify the dangers within used syringes including the range of illicit substances and non-viral microbes. By elucidating the narcotics and culturable organisms, this study provides unique insights to the health impacts of intravenous drug use among PWID.

### Current study

Drawing on the need to develop a more comprehensive set of knowledge about the real-world realities of the opioid crisis in a portion of the U.S. that has been disproportionately impacted by drug use and overdoses, the current study has three goals. First, we subject a sample of used drug needles obtained from PWID in Toledo, Ohio to forensic-based analyses to describe which narcotics, psychoactive compounds, and cutting agents are contained in the syringes. Second, we subject the contents of used drug syringes to a series of microbiological analyses to determine whether there are non-viral pathogens contained inside the syringes which were used for intravenous drug use. If pathogen(s) are detected, the third goal of this study is to identify sources of treatment for possible infections from those microbes. Since we are unaware of any other study which has simultaneously attempted to accomplish these goals, we treat this work as exploratory research and avoid making specific hypotheses about what may occur.

## Methods

### Obtaining the drug-used needles

Following a small pilot study in late 2022 and early 2023 where we obtained two dozen needles from the TLCHD in order to refine methodological practices used in this study (since the only purpose of the pilot study was to develop the procedures used in this study, we do not report any of these results), we obtained five containers of used needles – two one-quart sharps containers, one five-gallon sharps container, and two empty jugs of laundry detergent – from the TLCHD on November 8^th^ of 2023. Each one-quart sharps container was given to a household of PWID for use and returned full of used syringes. The five-gallon sharps container contained needles turned in to the TLCHD by PWID who visited the mobile harm reduction unit during week prior to collection of the syringes. The laundry detergent jugs – a common way of disposing of needles among PWID in Toledo – were turned in by households of PWID.

Due to the volume of needles that the TLCHD collects, the research team wanted to attempt to identify both narcotics and pathogens off of needles as they naturally exist in the population – in disposable containers (i.e., medical waste). As efforts to combat cross-contamination would have likely meant needing to perform ethically-challenging, multiple-step interactions with PWID by research team members (e.g., skin swabs prior to injection; sterile individual sharps containers designed for only one needle at a time; ensuring no needle sharing was taking place, etc.) in potentially hazardous environments, no efforts were made to stop cross-contamination between needles. Instead, this study was designed to meet the existing realities of the area where they were at in an attempt to learn more about intravenous drug use in the geographic region.

### Goal 1: Identifying the drugs and substances in syringes

#### Drug identification.

The first goal of this study was to determine which narcotics, psychoactive compounds, and cutting agents were contained in syringes used in intravenous drug use from Toledo, Ohio. Following securing the needles per established laboratory protocols, the research team randomly selected ten needles from each container for narcotic analysis (50 total). Next, the team sent the 50 syringe-derived narcotic samples to The Center for Forensic Science Research and Education (CFSRE) in Horsham, PA for forensic analysis to discover the narcotic-based compounds in each syringe. Methodologically, prior to sending the contents to the CFSRE, the plunger was removed from each syringe and 800 µL of 0.85% saline was added to the syringe and pushed through into a 1.7 mL Eppendorf tube. CFSRE aliquoted 100 µL of the sample into 900 microliters of mobile phase (95:5 10 mM Ammonium Formate in H2O: 0.1% formic acid in 50:50 MeOH:ACN) for analysis. Sample analysis was completed using a SCIEX TripleTOF ™ 5600 + quadrupole time-of-flight mass spectrometer (QTOF-MS) coupled with a Shimadzu Nexera XR ultra-high performance liquid chromatograph (LC). Chromatographic separation was achieved using a Phenomenex® Kinetex C18 analytical column (3.0 x 50 mm, 2.6 um) using gradient elution. Column temperature was 30ºC, flow rate was 0.4 mL/min, and injection volume was 5 uL. Positive electrospray ionization (ESI+) was used, and the final run time was 15.5 min. Samples were compared to a database containing over 1,200 drugs including novel psychoactive substances, traditional drugs, metabolites, adulterants, precursors, byproducts, and other esoteric drugs. The relative amount of each substance was determined by comparing the peak area of each of the compounds to the peak area of the main drug in the sample in the data. The main drug was selected through a review of the chromatogram. A list of the substances identified in the 50 drug-analyzed syringes and the microbes found in each of those syringes can be found in S1 Table.

### Goal 2: Identifying the microbes in syringes

#### Microbial isolation of syringe-derived microbes.

The second goal of this study was to process used drug needles from Toledo, Ohio to isolate and identify microbial content. To accomplish this goal methodologically, 103 syringes (the 50 used in drug identification + 53 other random used syringes from the same batch of used needles) were used to isolate microbes. To prevent sample contamination from microbes outside of the syringes, the needles were wiped with ethanol to sterilize prior to processing. From each used needle, the plunger was removed and 800 µL of 0.85% saline was added to the syringe and pushed through into a 1.7 mL Eppendorf tube. Aliquots of 100 µl was spread plated on tryptic soy agar (TSA, Becton Dickinson) and yeast peptone dextrose (YPD, 20% w/v peptone, 10% w/v yeast extract, 24% agar, 2% w/v dextrose) agar and incubated at 24°C for 48 hours. Colonies were streaked to isolation, and tryptic soy broth (TSB, Becton Dickinson) or YPD medium (as stated above without agar) was inoculated with each strain it was isolated on, incubated, and pure cultures were stored at stored at −80°C in medium with 20% v/v glycerol.

#### Microbial identification of syringe-derived strains.

In order to identify the microbes from syringes, gene sequencing of the small subunit rRNA gene sequencing and BLAST analysis was performed. For gene sequencing, colony PCR was used to amplify the small subunit rRNA gene [17]. Primers targeting the 16S (forward primer, 27F: 5′-AGR GTT TGA TCM TGG CTC A-3′; reverse primer, 1492R: 5′-TAC GGY TAC CTT GTT AYG ACT T −3′) and 18S rRNA gene (forward primer, nu-SSU-0777–5: 5′-AGAGTGTTCAAAGCAGGC-3′; reverse primer, nu-SSU-1648–3: 5′-ANCCATTCAATCGGTANT-3′) were used to amplify and sequence the locus. PCR conditions were [92°C denaturation for 10s, 50°C (16S) or 44.5°C (18S) annealing for 30s, elongation at 72°C for 90s] and repeated 30 times. Sanger sequencing was performed, using the 27F primer or nu-SSU-0777–5, by Genewiz. Strains were identified at the genus level through NCBI BLAST analysis of the sequence. A nucleotide alignment was generated from partial sequence of the 16S and 18S rRNA genes, and a maximum likelihood tree was constructed and edited using the CLC Main Workbench. *C. parapsilosis* strain CBS 604 18S rRNA gene was used as a reference sequence. GenBank accession numbers for the fungi 18 rRNA genes are PP316258-PP316285 and PQ097517-PQ097534. GenBank accession numbers for the bacterial 16 rRNA genes are PP316206-PP316237 and PQ112157 - PQ112258. *Candida* strains were identified at the species level by the Charles River Lab using MALDI-TOF mass spectrometry.

#### Growth and biofilm characteristics of an opportunistic pathogen.

In order to cause infection, human pathogens must be able to grow at 37°C, body temperature. *C. parapsilosis* strains n23, n30, n42, and n47 were tested for growth at 23°C and 37°C. The yeast were inoculated in 3 mL of YPD medium and incubated overnight with shaking at 24°C or 37°C. The culture was diluted 1:100 in RPMI-1640 + 2% Glucose + 0.165M MOPS media and adjusted to O.D._600_ of 0.2 to obtain approximately the same number of cells. 350 μL of the diluted cells were transferred to a single well in 96-well U-bottom microtiter plate (Costar REF 2797). The plates were incubated at 24°C or 37°C for 35 hours with continuous shaking and O.D._600_ was measured every 15 minutes using a BioTek Synergy HT plate reader. At least three replicates were performed.

The production of biofilm is also a characteristic of *Candida* yeast pathogens. We thus tested the syringe-derived yeast for biofilm formation using a 96-well plate assay [18]. *C. parapsilosis* strains n23, n30, n42, and n47 were inoculated in 3 mL of YPD medium and incubated overnight with shaking at 24°C or 37°C. The culture was diluted to 1:100 in RPMI-1640 (ThermoFisher Scientific) + 2% Glucose + 0.165M MOPS media and then adjusted to O.D._600_ of 0.2 to obtain approximately the same number of cells. 350 μL of the diluted cells were transferred to a single well in 96-well U-bottom microtiter plate (Costar REF 2797). The plates were incubated at 24°C or 37°C for 24 hours. After incubation, the cells and media were dumped out by inverting the plate. The plate was then rinsed with water to remove unbound cells. 125 μL of a 0.1% solution of crystal violet in water was added to each well containing cells and incubated at room temperature for 10–15 minutes. Crystal violet stains biofilm production. The wells were rinsed three times with water and then air-dried for four hours at room temperature. 125 μL of 30% acetic acid in water was added to each well and incubated for 15 minutes. The solubilized solution was transferred to a new flat bottomed 96-well microtiter plate (Falcon REF 351172) and absorbance was measured in a plate reader at 550 nm using 30% acetic acid in water as the blank. A BioTek Synergy HT plate reader was used to measure absorbance at 550 nm to identify biofilm formation. At least three replicates were performed.

### Goal 3: Identifying a potential antifungal agent for treatment

#### Antifungal drug discovery.

The third goal of this study was to provide insight for treatment, if needed, from possible infections of syringe-derived microbes. Since *C. parapsilosis* – an opportunistic fungus which causes infections for which few drugs are available for treatment – was identified, we sought to use soil-derived bacteria as a source of drug discovery for a new antifungal agent. Since the most effective antimicrobial compounds have been identified from soil microbes, soil is an ideal medium for the identification of bacteria that may have antifungal capacities. *Pseudomonas* stains, which are commonly found in soil, serve as a model organism for natural product discovery. If successful, these efforts could serve as an empirically-backed starting point for a drug discovery process.

To methodologically isolate soil-based bacteria, we collected samples of dirt from Bowling Green, Ohio – a town about 20 miles south of Toledo. To isolate the bacteria contained in each sample, one gram was suspended 5 mL 0.85% saline solution. A 10-fold serial dilution was performed and 100 µL aliquots were spread-plated on cetrimide agar medium to select for the growth of environmental *Pseudomonas* strains. Colonies were picked streaked for isolation on cetrimide agar medium to ensure pure cultures. All strains were subject to 16S gene sequencing and BLAST analysis as described above. GenBank accession numbers for *Pseudomonas* sequences are PP727019-PP727070. These strains were used to test for antagonistic activity.

#### Antagonistic plate assay.

Soil-derived environmental *Pseudomonas* strains were streaked to isolation on TSA and cultured for 20 hours at 23˚C. Pure colonies were inoculated in an individual well of a 96-well plate which contained 1 mL of tryptic soy broth. The yeast strain was streaked to isolation on YPD agar and cultured overnight at 30°C. An isolated colony was inoculated in liquid YPD medium and then cultured overnight at 30°C. To generate a confluent lawn of *Candida*, 100 µL of the overnight culture was spread on YPD agar plates. Subsequently, 1 µL of each environmental strain was transferred to the lawn using a 96-pin replicator (Boekel Microplate Replicator). Strains were co-cultured at 30°C for 24 hours and antagonistic activity was scored as positive for a given strain if a zone of clearing of at least 1 mm was produced on the yeast lawn surrounding the environmental strain. To confirm results, all inhibitory strains were selected and tested for activity at least three times. Strain TE3–3-F2023 was chosen for antifungal drug discovery since it inhibited multiple *Candida* pathogens and was amenable to transposon mutagenesis.

#### Genome sequencing and annotation of TE3–3-F2023.

*Pseudomonas* sp. TE3–3-F2023 bacterial genomic DNA was extracted using the Wizard DNA Purification Kit (Promega) and outsourced to the SeqCenter in Pittsburgh, PA for whole genome sequencing. Libraries were prepared according to the Oxford Nanopore Technology Ligation Sequencing kit specifications (SQK-LSK109). Quality control and adapter trimming was performed using porechop v.0.2.3 (https://github.com/rrwick/Porechop) and assembly statistics were performed with QUAST v.5.0.2 [19]. The genome was assembled using Flye v.2.9.1 [20] and annotated using Bakta [21] v.1.8.2. The *Pseudomonas* sp. TE3–3-F2023 genome sequence was submitted and annotated by Genbank and JGI IMG GOLD [22] and have corresponding accession number CP145113 and genome ID 8069812833, respectively.

#### Transposon mutagenesis.

Tn mutagenesis was performed, as previously described [23] in order to identify genes involved in antifungal production. Briefly, a 1 mL overnight culture of *Pseudomonas* sp. TE3–3-F2023 and *E. coli* strains HB101 and CC118 were centrifuged for 3 minutes at 13,000-rpm. The supernatant was removed, and the bacteria were each suspended in 500 µL 10 mM MgSO_4_. The *Pseudomonas* strain was heat shocked at 42°C for 30 minutes. 100 µL of the *Pseudomonas* strain was combined with 100 µL of HB101 and CC118. The triparental mating culture was vortexed and centrifuged for 3 minutes at 13,000-rpm. The supernatant was removed and the culture was resuspended in 10 µL of MgSO_4_. The cell mixture was spotted on TSA and incubated at 30°C for 24 hours. The spotted cell growth was scraped from the plate and reconstituted in 200 µL MgSO_4_ and diluted 10^−2^ in 20 mL of 0.85% NaCl. 100 µL aliquots were spread plated on a cetrimide agar medium with 50 µg/mL kanamycin to select for *Pseudomonas* transconjugants. Mutants were replica-plated onto *C. parapsilosis* n47and screened for a loss-of-antagonistic phenotype.

#### Mutant DNA extraction and arbitrary (ARB) PCR.

Genomic DNA was extracted from the mutant strain TE3–3-F2023 using the Wizard Genomic DNA Purification kit (Promega). ARB-PCR was used to amplify the genomic DNA flanking the Tn insert [24,25]. Briefly, two PCR cycles were performed. ARB-PCR I was performed using 2 μL of genomic DNA, and 5 μM primer ARB6 [GGCACGCGTCGACTAGTACNNNNNNNNNNACGCC], in combination with 5 μmol/L primer ME-I-extR [CTCGTTTCACGCTGAATATGGCTC] or 5 μmol/L primer ME-O-extF [CGGTTTACAAGCATAACTAGTGCGGC]. The conditions for ARB-PCR I reaction were [5 minutes at 95°C, six cycles of 30 seconds at 95°C, 30 seconds at 30°C and 90 seconds at 72°C, 30 cycles of 30 seconds at 95°C, 30 seconds at 45°C, and 90 seconds at 72°C, followed by an extension period of 4 minutes at 72°C]. For the second round of ARB-PCR, 1 μL of ARB-PCR I product was used as the template. ARB-PCR II was performed using 1 μL of ARB-PCR I product and 5 μmol/L primer ARB2 [GGCACGCGTCGACTAGTAC] in combination with 5 μmol/L primer ME-I-intR [CAGTTTTATTGTTCATGATGATATA] or 5 μmol/L primer ME-O-intF [AGAGGATCCCCGGGTACCGAGCTCG]. The conditions for ARB-PCR II reaction were [60 seconds at 95°C, followed by 30 cycles of 30 seconds at 95°C, 30 seconds at 52°C and 90 seconds at 72°C, followed by an extension period of 4 minutes at 72°C]. PCR purification was performed using ExoSAP-IT according to manufacturer recommendation (ThermoFisher Scientific) on each ARB-PCR II product. Samples were Sanger sequenced by Genewiz using either ME-I intR primer or ME-O intF primer.

## Results

### Goal 1: Identifying the drugs and substances in syringes

**Fig 1:**
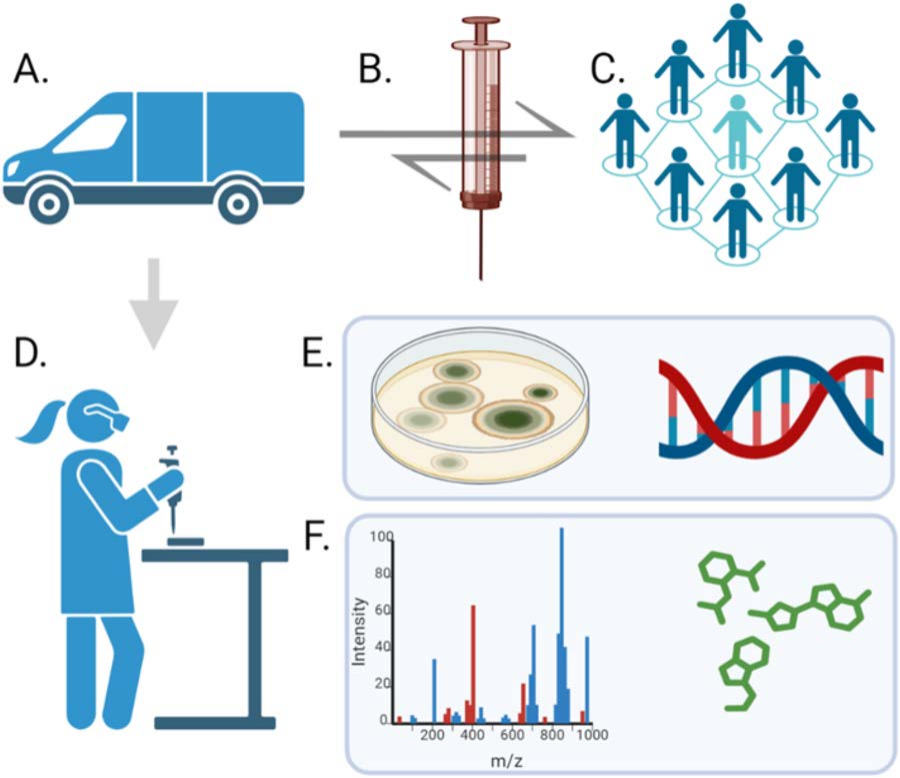
Workflow of microbial isolation and drug identification. (A) The TLCHD mobile unit provides a risk management program which offers (B) clean needles to (C) PWID. Arrows represent the exchange of clean and used needles. (D) Used needles are processed for (E) microbial isolation and identification, and (F) drug content by mass spectrometry.

The first goal of the study was to determine which narcotics, psychoactive compounds, and cutting agents were contained in the syringes collected from Toledo, Ohio. Using a multistep research design that involved several key entities and data collection points (Fig 1), 50 syringes used by PWID were collected in collaboration with the TLCHD and the NOSS program. The syringes were washed with saline solution and analyzed by liquid chromatography/time of flight mass spectrometry to identify drug contents and relative quantities (Table 1). The analytes were identified from a database containing approximately 1,200 compounds including controlled substances, pharmaceuticals, adulterants, and drug-related compounds.

**Table 1:** Results of substances and parts contained in analyzed drug-used syringes (n = 50 syringes).

General Descriptives				
μ number of substances in syringes	7.800			
SD around μ	3.270			
Min. substances in a syringe	1			
Max. substances in a syringe	15			
	**In How Many Syringes (%)**	***Parts Analysis***		
**Substance**		**Parts μ**	**Parts SD**	**Parts Range**
Fentanyl	98%	0.983	0.121	0.150–1.000
Cocaine	90%	0.754	1.436	0.020–8.810
Xylazine	86%	5.577	6.104	0.010–26.100
4-ANPP	84%	0.280	0.137	0.060–0.740
Quinine/Quinidine	84%	0.615	0.742	0.010–3.740
Diphenhydramine	64%	0.302	0.356	0.040–1.280
6MAM	38%	0.179	0.222	0.010–0.700
Acetyl Fentanyl	34%	0.013	0.007	0.010–0.030
Norfentanyl	32%	0.017	0.009	0.010–0.040
Caffeine	28%	0.126	0.115	0.010–0.330
Ethyl 4-ANPP	22%	0.024	0.020	0.010–0.060
Morphine	20%	0.133	0.150	0.010–0.510
Lidocaine	18%	0.068	0.073	0.010–0.230
Tramadol	16%	0.083	0.119	0.010–0.430
N-propionyl Norfentanyl	14%	0.010	0.000	0.010–0.010
Acetaminophen	12%	0.017	0.005	0.010–0.020
Fluorofentanyl	6%	0.073	0.042	0.040–0.120
Medetomidine	6%	0.010	0.000	0.010–0.010
Acetylcodeine	4%	0.030	0.014	0.020–0.040
Heroin	4%	0.015	0.007	0.010–0.020
Phenethyl 4-ANPP	4%	0.015	0.007	0.010–0.020
Codeine	2%	0.060	--^A^	-- ^A^
Despropionyl para-fluorofentanyl	2%	0.020	-- ^A^	-- ^A^
Isotonitazene	2%	0.010	-- ^A^	-- ^A^
Papaverine	2%	0.020	-- ^A^	-- ^A^
Quetiapine	2%	0.010	-- ^A^	-- ^A^
Trazodone	2%	0.010	-- ^A^	-- ^A^

μ = mean; SD = standard deviation.

^A^ The SD and range are incalculable due to only being identified in one used syringe.

The average drug-used syringe contained nearly eight substances (μ = 7.800; SD = 3.280; range = 1–15), suggesting that polysubstance cocktails may be the norm for the population of PWID in the greater Toledo area from 2023–2024 (a finding corroborated by local [11] and national reports [16]). Most of the 27 products found in the needles were drugs listed in the Drug Enforcement Administration’s (DEA’s) schedule of controlled substances. Most needles contained opioids (e.g., fentanyl), stimulants (e.g., cocaine), and sedatives (e.g., xylazine) in varying combinations. An analysis of the contents of parts in the needles demonstrates that xylazine (μ = 5.577), fentanyl (μ = 0.98 [commonly the anchor for parts analysis in these data]), cocaine (μ = 0.754), and quinine (μ = 0.615) are the most common substances contained in the needles in terms of volume. In addition to the ubiquitous presence of fentanyl, high standard deviations demonstrate that several needles contain alarmingly high levels of xylazine (SD = 6.104) and cocaine (SD = 1.436). The common presence of stimulants may indicate that drug dealers and/or PWID were purposefully mixing drug cocktails to keep users alert in the face of a slew of opioids (e.g., fentanyl, acetyl fentanyl) which carry an increased risk of fatal overdose via pulmonary depression. The presence of xylazine also contributes to overdoses by depressing the central nervous system but is difficult to identify in clinical settings, as effects often appear similar to opioids and may not be included in routine drug screening tests [26]. Xylazine also causes skin necrosis which results in dead tissue and open wounds [27] which has potential for bacterial infections and may lead to limb amputation. The presence of xylazine is relatively new to the drug community (26).

### Goal 2: Identifying the microbes in syringes

**Fig 2:**
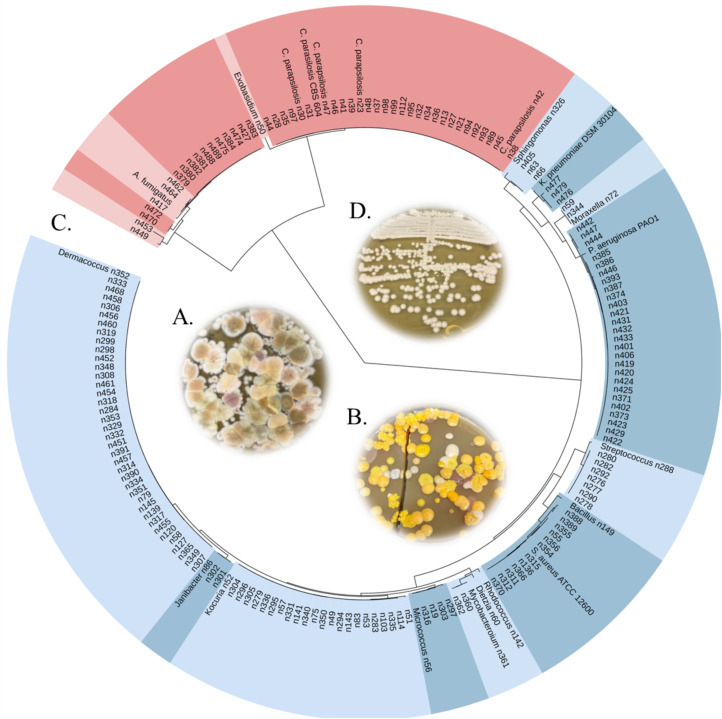
Microbes isolated from syringes. A subset of the (A) fungi and (B) bacteria isolated from used syringes on YPD and TSA plates, respectively. (C) A phylogenetic tree was constructed based on the 16S or 18S rRNA gene. Bacterial and fungal clades were highlighted shades of blue and pink, respectively, and the genus of each clade was identified. Potential fungal pathogens consist of *C. parapsilosis* strains*.* (D) *C. parapsilosis* n47 on YPD agar.

The second goal of this study was to isolate microbes from syringes and determine if pathogens were present. To achieve this goal, syringe contents of the 50 used drug needles and an additional 53 random syringes from the same containers (a total of 103 needles) were processed and spread on tryptic soy agar (TSA) and yeast peptone dextrose (YPD) plates with and without cycloheximide. Growth was observed on all plates (Fig 2A and 2B). To identify syringe-derived culturable microbes, 180 colony forming units were picked from 103 used syringes (including the 50 syringes discussed above used in drug identification; Table S1). NCBI BLAST analysis of the small subunit ribosomal rRNA gene was used to determine the genus of the strains, and relatedness was visualized through phylogenetics (Fig 2C); 131 and 49 were of bacterial and fungal origin, respectively. Most of the bacteria represent known members of the skin flora [28], and have been associated with infections among users [29,30]. The identified eukaryotic strains consisted of three *Aspergillus*, 30 *Candida*, two *Cladosporium*, one *Exobasidium*, 10 *Penicillium*, and three *Yarrowia*. Thirty of the fungi consisted of *Candida* and strains n23, n30, n42, and 47 (Fig 2D) were identified as *C. parapsilosis* and the two *Aspergillus* strains n462 and n464 as *A. versicolor* using MALTI-TOF mass spectrometry analysis. Phylogenetics suggested all *Candida* isolates were *C. parapsilosis*, which is an emerging fungal pathogen [31,32], and most were isolated from different syringes, thus suggesting that it may be common in drug-used needles. Indeed, life-threatening *Candida* infections have been observed among PWID [33–36], and the World Health Organization (WHO) recently released a fungal priority pathogen list in 2022 to guide research, development, and public health action [37]. Four *Candida* species, including *C. parapsilosis*, were noted in the WHO list because these organisms are increasingly causing infections [38,39] and outbreaks [40–43] and have evolved resistance to the few anti-fungal agents that are available for treatment [44,45].

To determine if the four syringe-derived *C. parapsilosis* strains showed pathogenic traits, a growth curve analysis and biofilm assays were performed. Results revealed that a 37°C was preferred over 24°C (S1 Fig), and all isolates also exhibited biofilm activity (S2 Table), thus expressing factors that contribute to survival within the human host. While other virulence factors are also required for disease, these yeasts present a potential threat of infection. Unfortunately, only three classes of antifungal agents are used to treat yeast infections [46], and most *Candida* exhibit resistance to azoles, which have been applied in agriculture, and some resistance has also been observed with echinocandins and amphotericin B [47], the standard treatment for yeast infections. Evolved resistance to these drugs would result in untreatable and possibly deadly infections.

### Goal 3: Identifying a potential antifungal agent for treatment

**Fig 3:**
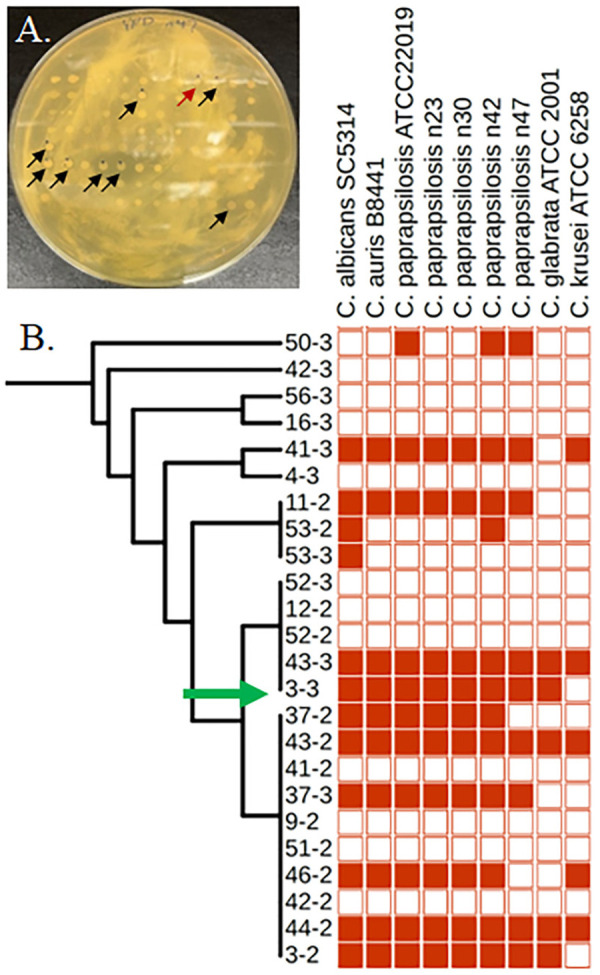
Antagonistic activity of *Pseudomonas* strains against *Candida* species. (A) An antagonistic plate assay was used to identify bacteria that could inhibit the growth of *Candida*. Ninety-six environmental bacteria were stamped onto the spread plated *C. parapsilosis* n47 strain. A zone of clearing around a bacterial colony indicated inhibition (indicated by arrows). The red arrow indicates activity from *Pseudomonas* strain 3−3. (B) A phylogenetic tree of 24 closely related soil-derived environmental isolates based on the 16S rRNA gene. *Pseudomonas* strains were depicted on the left y-axis and *Candida* across the top x-axis. Red squares indicate specific inhibition of *Candida* strain.

Since the need for new antifungal agents is a pressing global need (*39*), the third goal of this study investigated if 96 soil-derived bacteria strains were capable of inhibiting the growth of different *Candida* species (Fig 3A). In addition to the four syringe-derived *C. parapsilosis* isolates, antagonistic assays were also performed with five reference *Candida* strains including *C. albicans* SC5314, *C. auris* B8441, *C. parapsilosis* ATCC 22019, *C. glabrata* ATCC 2001, and *C. krusei* ATCC 6258. Antagonistic activity was observed among 13 of 84 environmental *Pseudomonas* strains (Fig 3B). In total, 84 antagonistic events were identified from 864 individual competitions consisting of 96 soil-derived bacteria and nine *Candida* strains. Nine *Pseudomonas* strains antagonized at least seven of the *Candida* indicating the remarkable ability of these bacteria to inhibit the growth of yeast. As a group, *Pseudomonas* are known to be diverse and produce a range of compounds involved in a variety of applications [48,49]. To gain insight into the antagonistic factor, transposon mutagenesis was optimized with strain TE3–3-F2023, which inhibited seven *Candida* strains, to identify genes involved in anti-fungal activity. More than 10,000 mutants were screened and eight were found with a loss-of-killing phenotype.

**Fig 4:**
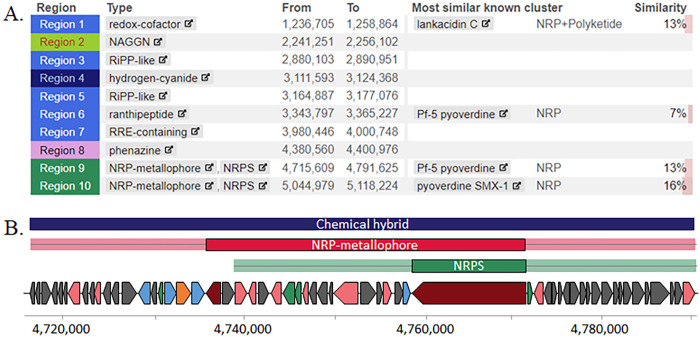
The BGC identified in wild-type *Pseudomonas* strain 3−3 that was involved in antagonistic activity. (A) antiSMASH results of predicted BGCs in strain 3−3. (B) Region 9 was identified as the BGC involved in antagonistic activity. The gene cluster was 76 kb and predicted to encode a metallophore (red and light red box) and a NRPS (green and light green box)-derived product. The open reading frames involved in production and secretion of the product were shaded according to function: core biosynthetic genes, maroon; additional biosynthetic genes, pink; transport-related genes, light blue; regulatory genes, green; resistance genes, orange; other genes, grey.

Of the 10 biosynthetic gene clusters identified by antiSMASH [50] (Fig 4A), one was disrupted by the transposon in two independent mutants, indicating those loci were involved in antagonistic activity (Fig 4B). The predicted product may represent a non-ribosomal peptide metallophore which links a metal ion binding protein to an antibiotic [51]. Other loci were also discovered whose products were predicted to play a role in the expression, modification, and transport of the anti-fungal compound (S3 Table). A BLAST analysis of the gene cluster showed that 42 isolates in the NCBI database, which were all *Pseudomonas*, exhibited comparable genetic content (96% query coverage and 89% nucleotide similarity) suggesting other strains may also produce this product. However, we believe this is the first reported study that identifies a gene cluster possibly involved in metallophore production whose product is active against yeast, thus providing evidence for a new source of anti-microbial compounds against emerging fungal pathogens.

## Discussion

In collaboration with the TLCHD, this study analyzed used drug needles to accomplish three goals: First, what narcotics were contained in the needles? Our results demonstrated that there were, on average, almost eight psychoactive substances contained in a typical syringe. Second, are there dangerous, emergent, opportunistic non-viral pathogens on the needles? After the identification of a series of dangerous pathogens, including the fungus called *Candida parapsilosis* which causes potentially fatal infections*,* the answer to this was a resounding ‘yes.’ Third, can bacteria be identified which inhibits the growth of the dangerous pathogens? Indeed, certain soil-derived bacteria do have the ability to kill *Candida parapsilosis*, thereby potentially forming a empirically-backed starting point for a drug discovery process to treat people who have been infected with *Candida parapsilosis*.

If corroborated by other studies, our findings present initial evidence that highlights a somewhat scary possibility: The dynamic and dangerous world of intravenous drug use may hold unrealized dangers – dangers like medicinally resistant microbes that carry the ability to cause deadly infections. In addition to the mixture of various drugs that pose a high risk of fatal overdose (Table 1), xylazine was identified which was listed by the DEA in 2021 as a new national threat [52] and previously thought to be localized to large coastal cities such Los Angeles and Philadelphia [53]. In addition to the illicit drugs, opportunistic non-viral pathogens were also present. While understanding these different risks associated with intravenous drug use may be more serious than previously expected, results are limited by sample size. First, only 50 used drug needles were analyzed for both narcotics and pathogens and 53 additional needles for microbes alone (S1 Table) – a number which pales in comparison to the 284,123 needles distributed by the TLCHD NOSS program in 2023. Despite analyzing only a fraction of these, opportunistic pathogens not previously found in syringes were identified, and additional pathogens are likely present in needles based on the odds of probability in the Toledo-area population. Second, while these needles came from one of the most narcotic-impacted areas in the U.S., the data are derived from only a single location. Many other state locales have also been detrimentally impacted by drug use, and results likely vary based on location, time, and space. Moreover, regional variations in climate and carbon source from the narcotics and cutting agents may impact microbial growth [54] and the effects of drugs [55], meaning that these conclusions are not nationally representative. Third, less than 1% of microbes can be cultured from the environment, so this analysis only identified a few of the organisms that are likely present in used needles.

Although we cannot determine whether the syringes analyzed were shared among multiple people, the identification of many constituents suggests that needle sharing is a possibility [56]. Regardless, results highlight the extreme risk of fatal overdose for street drugs in the Toledo area – a threat that is corroborated both these data and other recent studies [26,57]. While the dangerous mixtures found in the syringes pose a public health risk on their own, the intravenous drug-using environment itself fosters unhealthy behaviors and hygiene practices that promote infections [58,59]. Microbes present in used needles that originate from skin flora or as contaminants from the environment or manufactured drugs may also be life-threatening (30). Thus, infections from opportunistic pathogens like *Candida* might pose a real risk in ways not previously discovered (Fig 2).

Previous work suggests that *Candida* may be evolving from an environmental strain towards a pathogenic variant in humans [60,61], and continuous exposure to humans through shared needles may further select for pathogenic traits. Furthermore, *Candida* infections [62,63] and hospital outbreaks [40–43] are increasing among those who do not inject drugs, suggesting evolutions towards pathogenicity. Unfortunately, pathogenic *Candida* are already evolving resistance to the few antifungal drugs available for treatment [46,47,64], so the discovery of new therapeutics is critical. We identified a biosynthetic gene cluster that was predicted to encode a non-ribosomal peptide derived metallophore (Fig 3).

Metals and their respective binding proteins, such as iron and siderophores, are sequestered by bacteria because those metals are required for growth [65]. Metallo-antibiotic compounds are described as a Trojan Horse complex since cheating bacteria import these products, produced by other bacteria, for survival yet are inhibited or killed by the attached drug [66]; these compounds represent a promising new treatment for antibiotic resistant infections. One recently FDA approved metallo-antibiotic, Cefiderocol, is active against multi-drug and carbapenem-resistant Gram-negative bacterial pathogens [67,68]. *Pseudomonas* are known to produce over 100 different types of metal binding proteins [69,70], making this group an ideal candidate for drug discovery.

Altogether, this work provides new insight to the ever-changing opioid crisis by highlighting previously undiscovered dangers that could be associated with intravenous drug use. If confirmed by other scholars using more expansive data collection practices across larger geographic strata, the findings have potential to improve treatment for PWID, facilitate antifungal drug discovery, and to provide pertinent information to clinicians, researchers, and public health officials tasked with combatting the opioid crisis. Concurrent nationwide studies are needed to provide insight to the challenges that other cities and rural areas encounter in order to comprehend the influx of drugs and treatments that are needed.

## Supporting information

S1 Table Identified substances and microbes from 50 used syringes. (DOCX)

S2 Table Biofilm formation of *C. parapsilosis* isolates. (DOCX)

S3 Table Genes identified in loss-of-killing mutants. (DOCX)

S1 Fig Grow curve analysis of *C. parapsilosis* strains (A) n23, (B) n30, (C) n42, and (D) n47 at 24°C (gray) and 37°C (red). (TIF)
