# Supplementary material for: Opportunistic pathogens and polycocktail drugs fuel dynamic public health threats during the opioid crisis
Source: PLoS One. 2025 Aug 12;20(8):e0326200. doi: 10.1371/journal.pone.0326200 (PMC12342250; doi:10.1371/journal.pone.0326200)
Supplement: S1 Table — (DOCX) [file pone.0326200.s001.docx]

| S1 Table. Identified substances and microbes from 50 used syringes. | | | |
| --- | --- | --- | --- |
| **Syringe** | **Drugs (ppm)** | **Microbes (Genus and strain number)** | |
| 1 | Xylazine (6.71) | *Streptococcus* | 276 |
|  | Fentanyl (1) | *Streptococcus* | 277 |
|  | Quinine/Quinidine (0.45) | *Streptococcus* | 278 |
|  | 6MAM (0.44) | *Kocuria* | 279 |
|  | Cocaine (0.24) |  |  |
|  | 4-ANPP (0.24) |  |  |
|  | Diphenhydramine (0.23) |  |  |
|  | Lidocaine (0.07) |  |  |
|  | Acetylcodeine (0.04) |  |  |
|  | Morphine (0.04) |  |  |
|  | Acetyl Fentanyl (0.03) |  |  |
|  | Tramadol (0.03) |  |  |
|  | Papaverine (0.02) |  |  |
|  | Caffeine (0.01) |  |  |
| 2 | Xylazine (12.02) | *Streptococcus* | 288 |
|  | Quinine/Quinidine (2.09) | *Streptococcus* | 280 |
|  | Cocaine (1.17) | *Streptococcus* | 282 |
|  | Fentanyl (1) | *Kocuria* | 283 |
|  |  | *Dermacoccus* | 284 |
| 3 | Xylazine (9.41) |  |  |
|  | Quinine/Quinidine (3.74) |  |  |
|  | Cocaine (2.62) |  |  |
|  | Fentanyl (1) |  |  |
|  | 6MAM (0.44) |  |  |
|  | 4-ANPP (0.22) |  |  |
|  | Morphine (0.17) |  |  |
| 4 | Xylazine (2.70) |  |  |
|  | Fentanyl (1) |  |  |
|  | Cocaine (0.56) |  |  |
|  | Quinine/Quinidine (0.52) |  |  |
| 5 | Xylazine (26.10) |  |  |
|  | Cocaine (1.74) |  |  |
|  | Fentanyl (1) |  |  |
|  | Quinine/Quinidine (0.92) |  |  |

| Supplemental Table 1 (Continued). | | | |
| --- | --- | --- | --- |
| 6 | Xylazine (2.72) |  |  |
|  | Fentanyl (1) |  |  |
|  | Cocaine (0.64) |  |  |
|  | Quinine/Quinidine (0.32) |  |  |
|  | 4-ANPP (0.24) |  |  |
|  | Diphenhydramine (0.12) |  |  |
|  | Tramadol (0.10) |  |  |
|  | Lidocaine (0.08) |  |  |
|  | Caffeine (0.01) |  |  |
|  | Norfentanyl (0.01) |  |  |
|  | 6MAM (0.01) |  |  |
|  | Acetyl Fentanyl (0.01) |  |  |
|  | Ethyl 4-ANPP (0.01) |  |  |
| 7 | Xylazine (6.23) | *Micrococcus* | 303 |
|  | Fentanyl (1) | *Micrococcus* | 297 |
|  | Quinine/Quinidine (0.22) | *Kocuria* | 294 |
|  |  | *Kocuria* | 295 |
|  |  | *Kocuria* | 305 |
|  |  | *Kocuria* | 296 |
|  |  | *Kocuria* | 304 |
|  |  | *Janibacter* | 302 |
|  |  | *Janibacter* | 301 |
|  |  | *Dermacoccus* | 298 |
|  |  | *Dermacoccus* | 299 |
| 8 | Xylazine (14.29) | *Dermacoccus* | 307 |
|  | Cocaine (3.01) | *Dermacoccus* | 308 |
|  | Fentanyl (1) | *Dermacoccus* | 306 |
|  | 4-ANPP (0.24) |  |  |
|  | Quinine/Quinidine (0.21) |  |  |

| Supplemental Table 1 (Continued). | | | |
| --- | --- | --- | --- |
| 9 | Xylazine (11.91) |  |  |
|  | Fentanyl (1) |  |  |
|  | 6MAM (0.56) |  |  |
|  | Morphine (0.51) |  |  |
|  | Quinine/Quinidine (0.42) |  |  |
|  | 4-ANPP (0.32) |  |  |
|  | Diphenhydramine (0.25) |  |  |
|  | Lidocaine (0.13) |  |  |
|  | Tramadol (0.11) |  |  |
|  | Cocaine (0.07) |  |  |
|  | Codeine (0.06) |  |  |
|  | Acetyl Fentanyl (0.03) |  |  |
|  | Caffeine (0.03) |  |  |
|  | Acetylcodeine (0.02) |  |  |
|  | Norfentanyl (0.02) |  |  |
| 10 | Xylazine (12.89) | *Staphylococcus* | 311 |
|  | Fentanyl (1) | *Staphylococcus* | 312 |
|  | Quinine/Quinidine (0.92) |  |  |
|  | 6MAM (0.70) |  |  |
|  | 4-ANPP (0.48) |  |  |
| 11 | Cocaine (1) |  |  |
|  | Fentanyl (0.15) |  |  |
| 12 | Xylazine (12.04) |  |  |
|  | Fentanyl (1) |  |  |
|  | Quinine/Quinidine (0.50) |  |  |
|  | Cocaine (0.29) |  |  |
| 13 | Xylazine (8.30) | *Staphylococcus* | 315 |
|  | Fentanyl (1) | *Micrococcus* | 316 |
|  | Cocaine (0.87) | *Dermacoccus* | 318 |
|  | Quinine/Quinidine (0.46) | *Dermacoccus* | 319 |
|  | 6MAM (0.27) | *Dermacoccus* | 314 |
|  | 4-ANPP (0.20) | *Dermacoccus* | 317 |
| 14 | Xylazine (17.28) |  |  |
|  | Fentanyl (1) |  |  |
|  | Cocaine (0.95) |  |  |
|  | Quinine/Quinidine (0.82) |  |  |
|  | 6MAM (0.42) |  |  |
|  | Morphine (0.22) |  |  |
|  | 4-ANPP (0.17) |  |  |
|  | Diphenhydramine (0.14) |  |  |

| Supplemental Table 1 (Continued). | | | |
| --- | --- | --- | --- |
| 15 | Xylazine (4.06) |  |  |
|  | Fentanyl (1) |  |  |
|  | 4-ANPP (0.25) |  |  |
|  | 6MAM (0.16) |  |  |
|  | Quinine/Quinidine (0.12) |  |  |
|  | Diphenhydramine (0.07) |  |  |
|  | Ethyl 4-ANPP (0.05) |  |  |
|  | Morphine (0.03) |  |  |
|  | Lidocaine (0.02) |  |  |
|  | Cocaine (0.02) |  |  |
|  | Tramadol (0.01) |  |  |
|  | Acetyl Fentanyl (0.01) |  |  |
| 16 | Xylazine (10.56) |  |  |
|  | Fentanyl (1) |  |  |
|  | Quinine/Quinidine (0.43) |  |  |
|  | Diphenhydramine (0.17) |  |  |
|  | Cocaine (0.13) |  |  |
|  | 4-ANPP (0.12) |  |  |
|  | Morphine (0.11) |  |  |
| 17 | Xylazine (7.38) | *Sphingomonas* | 326 |
|  | Fentanyl (1) |  |  |
|  | Quinine/Quinidine (0.93) |  |  |
| 18 | Xylazine (19.22) | *Kocuria* | 331 |
|  | Fentanyl (1) | *Dermacoccus* | 332 |
|  | Tramadol (0.43) | *Dermacoccus* | 329 |
|  | 4-ANPP (0.27) | *Dermacoccus* | 333 |
|  | Lidocaine (0.23) |  |  |
|  | Cocaine (0.18) |  |  |
|  | Fluorofentanyl (0.12) |  |  |
|  | Quinine/Quinidine (0.11) |  |  |
|  | Morphine (0.04) |  |  |
| 19 | Xylazine (12.09) | *Kocuria* | 335 |
|  | Cocaine (2.32) | *Dermacoccus* | 334 |
|  | Fentanyl (1) |  |  |
|  | Quinine/Quinidine (0.55) |  |  |
|  | 4-ANPP (0.18) |  |  |
|  | 6MAM (0.17) |  |  |
|  | Diphenhydramine (0.14) |  |  |

| Supplemental Table 1 (Continued). | | | |
| --- | --- | --- | --- |
| 20 | Cocaine (8.81) | *Kocuria* | 340 |
|  | Xylazine (5.86) |  |  |
|  | Fentanyl (1) |  |  |
|  | Quinine/Quinidine (0.89) |  |  |
|  | 4-ANPP (0.20) |  |  |
|  | Diphenhydramine (0.18) |  |  |
|  | 6MAM (0.06) |  |  |
| 21 | Cocaine (1) | *Moraxella* | 344 |
| 22 | Xylazine (4.86) | *Kocuria* | 350 |
|  | Fentanyl (1) | *Dermacoccus* | 349 |
|  | Quinine/Quinidine (0.45) | *Dermacoccus* | 348 |
|  | Cocaine (0.16) | *Dermacoccus* | 352 |
|  | 4-ANPP (0.13) | *Dermacoccus* | 353 |
|  | Diphenhydramine (0.13) | *Dermacoccus* | 351 |
|  | Tramadol (0.06) |  |  |
|  | Morphine (0.05) |  |  |
|  | Lidocaine (0.04) |  |  |
| 23 | Xylazine (3.09) | *Staphylococcus* | 355 |
|  | Quinine/Quinidine (1.31) | *Staphylococcus* | 356 |
|  | Fentanyl (1) | *Staphylococcus* | 354 |
|  | Cocaine (0.34) |  |  |
|  | 4-ANPP (0.29) |  |  |
|  | Diphenhydramine (0.04) |  |  |
|  | Fluorofentanyl (0.04) |  |  |
|  | Tramadol (0.03) |  |  |
| 24 | Xylazine (5.49) |  |  |
|  | Fentanyl (1) |  |  |
|  | Quinine/Quinidine (0.20) |  |  |
|  | Morphine (0.15) |  |  |
|  | Diphenhydramine (0.12) |  |  |
|  | 4-ANPP (0.10) |  |  |
|  | Cocaine (0.04) |  |  |
| 25 | Xylazine (2.55) | *Pseudomonas* | 371 |
|  | Cocaine (1.14) | *Staphylococcus* | 366 |
|  | Fentanyl (1) | *Staphylococcus* | 370 |
|  | 4-ANPP (0.74) | *Mycobacteroium* | 361 |
|  | Fluorofentanyl (0.06) | *Mycobacteroium* | 360 |
|  | Norfentanyl (0.04) | *Mycobacteroium* | 362 |
|  | Acetaminophen (0.02) | *Kocuria* | 336 |
|  | Quinine/Quinidine (0.02) | *Dermacoccus* | 365 |
|  | 6MAM (0.02) |  |  |
|  | Despropionyl para-fluorofentanyl (0.02) |  |  |
| Supplemental Table 1 (Continued). | | | |
| 26 | Cocaine (1.59) | *Pseudomonas* | 374 |
|  | Fentanyl (1) | *Pseudomonas* | 373 |
|  | 4-ANPP (0.27) |  |  |
|  | Xylazine (0.25) |  |  |
|  | Diphenhydramine (0.12) |  |  |
|  | Quinine/Quinidine (0.08) |  |  |
|  | Phenethyl 4-ANPP (0.02) |  |  |
|  | Lidocaine (0.01) |  |  |
| 27 | Fentanyl (1) | *Pseudomonas* | 385 |
|  | 4-ANPP (0.24) | *Pseudomonas* | 386 |
|  | Diphenhydramine (0.14) | *Pseudomonas* | 393 |
|  | Cocaine (0.02) | *Pseudomonas* | 387 |
|  | Norfentanyl (0.02) | *Staphylococcus* | 388 |
|  | Acetyl Fentanyl (0.01) | *Staphylococcus* | 389 |
|  | Ethyl 4-ANPP (trace) | *Dermacoccus* | 390 |
|  |  | *Dermacoccus* | 391 |
|  |  | *Penicillium* | 381 |
|  |  | *Penicillium* | 380 |
|  |  | *Penicillium* | 382 |
|  |  | *Penicillium* | 379 |
|  |  | *Penicillium* | 384 |
|  |  | *Penicillium* | 383 |
| 28 | Xylazine (2.44) | *Sphingobium* | 405 |
|  | Fentanyl (1) | *Pseudomonas* | 419 |
|  | 4-ANPP (0.38) | *Pseudomonas* | 403 |
|  | Diphenhydramine (0.25) | *Pseudomonas* | 401 |
|  | Cocaine (0.22) | *Pseudomonas* | 406 |
|  | Quinine/Quinidine (0.05) | *Pseudomonas* | 402 |
|  | 6MAM (0.03) | *Aspergillus* | 417 |
|  | Norfentanyl (0.02) |  |  |
|  | Acetaminophen (0.02) |  |  |
|  | Ethyl 4-ANPP (0.02) |  |  |
|  | Acetyl Fentanyl (0.01) |  |  |

| Supplemental Table 1 (Continued). | | | |
| --- | --- | --- | --- |
| 29 | Fentanyl (1) | *Pseudomonas* | 429 |
|  | Cocaine (0.44) | *Pseudomonas* | 422 |
|  | 4-ANPP (0.36) | *Pseudomonas* | 421 |
|  | Diphenhydramine (0.11) | *Pseudomonas* | 431 |
|  | Xylazine (0.08) | *Pseudomonas* | 420 |
|  | Heroin (0.02) | *Pseudomonas* | 424 |
|  | Ethyl 4-ANPP (0.02) | *Pseudomonas* | 425 |
|  | Acetyl Fentanyl (0.01) | *Pseudomonas* | 423 |
|  | 6MAM (0.01) | *Penicillium* | 427 |
|  | Norfentanyl (0.01) |  |  |
|  | Quinine/Quinidine (trace) |  |  |
| 30 | Fentanyl (1) | *Pseudomonas* | 432 |
|  | Diphenhydramine (0.06) | *Pseudomonas* | 433 |
|  | Ethyl 4-ANPP (0.06) |  |  |
|  | 4-ANPP (0.06) |  |  |
|  | Xylazine (0.06) |  |  |
|  | Cocaine (0.05) |  |  |
|  | Quinine/Quinidine (0.04) |  |  |
|  | Acetyl Fentanyl (0.01) |  |  |
|  | Phenethyl 4-ANPP (0.01) |  |  |
|  | Norfentanyl (0.01) |  |  |
|  | Caffeine (0.01) |  |  |
|  | N-propionyl Norfentanyl (trace) |  |  |
| 31 | Fentanyl (1) | *Pseudomonas* | 442 |
|  | 4-ANPP (0.34) |  |  |
|  | Diphenhydramine (0.12) |  |  |
|  | Cocaine (0.04) |  |  |
|  | Xylazine (0.01) |  |  |
|  | Acetyl Fentanyl (0.01) |  |  |
|  | N-propionyl Norfentanyl (trace) |  |  |
| 32 | Fentanyl (1) |  |  |
|  | 4-ANPP (0.50) |  |  |
|  | Cocaine (0.02) |  |  |
|  | Acetyl Fentanyl (0.02) |  |  |
|  | N-propionyl Norfentanyl (0.01) |  |  |
|  | Norfentanyl (0.01) |  |  |
| 33 | Fentanyl (1) |  |  |
|  | 4-ANPP (0.19) |  |  |
|  | Cocaine (0.02) |  |  |
|  | Acetyl Fentanyl (0.01) |  |  |
|  | N-propionyl Norfentanyl (trace) |  |  |
|  | Ethyl 4-ANPP (trace) |  |  |
|  | Norfentanyl (trace) |  |  |

| Supplemental Table 1 (Continued). | | | |
| --- | --- | --- | --- |
| 34 | Fentanyl (1) |  |  |
|  | 4-ANPP (0.44) |  |  |
|  | Cocaine (0.05) |  |  |
|  | Acetyl Fentanyl (0.01) |  |  |
|  | Xylazine (0.01) |  |  |
|  | Norfentanyl (0.01) |  |  |
|  | N-propionyl Norfentanyl (0.01) |  |  |
| 35 | Fentanyl (1) |  |  |
|  | 4-ANPP (0.41) |  |  |
|  | Cocaine (0.24) |  |  |
|  | Diphenhydramine (0.16) |  |  |
|  | Xylazine (0.15) |  |  |
|  | Quinine/Quinidine (0.03) |  |  |
|  | Caffeine (0.01) |  |  |
|  | Acetyl Fentanyl (0.01) |  |  |
|  | 6MAM (0.01) |  |  |
|  | N-propionyl Norfentanyl (trace) |  |  |
|  | Norfentanyl (trace) |  |  |
| 36 | Xylazine (2.47) |  |  |
|  | Fentanyl (1) |  |  |
|  | Quinine/Quinidine (0.72) |  |  |
|  | Diphenhydramine (0.66) |  |  |
|  | Cocaine (0.61) |  |  |
|  | Caffeine (0.26) |  |  |
|  | 4-ANPP (0.12) |  |  |
| 37 | Fentanyl (1) | *Pseudomonas* | 447 |
|  | Quinine/Quinidine (0.78) | *Pseudomonas* | 444 |
|  | Diphenhydramine (0.69) | *Pseudomonas* | 446 |
|  | Cocaine (0.56) | *Yarrowia* | 449 |
|  | Xylazine (0.34) |  |  |
|  | Caffeine (0.18) |  |  |
|  | 4-ANPP (0.16) |  |  |

| Supplemental Table 1 (Continued). | | | |
| --- | --- | --- | --- |
| 38 | Fentanyl (1) |  |  |
|  | Diphenhydramine (0.88) |  |  |
|  | Quinine/Quinidine (0.88) |  |  |
|  | Xylazine (0.46) |  |  |
|  | 4-ANPP (0.33) |  |  |
|  | Caffeine (0.28) |  |  |
|  | Cocaine (0.24) |  |  |
|  | Tramadol (0.02) |  |  |
|  | Lidocaine (0.01) |  |  |
|  | Ethyl 4-ANPP (0.01) |  |  |
|  | Acetaminophen (0.01) |  |  |
|  | Medetomidine (0.01) |  |  |
|  | Acetyl Fentanyl (0.01) |  |  |
| 39 | Xylazine (5.80) |  |  |
|  | Fentanyl (1) |  |  |
|  | 4-ANPP (0.46) |  |  |
|  | Quinine/Quinidine (0.22) |  |  |
|  | Diphenhydramine (0.06) |  |  |
|  | Caffeine (0.06) |  |  |
|  | Tramadol (0.04) |  |  |
|  | Acetaminophen (0.02) |  |  |
|  | Medetomidine (0.01) |  |  |
|  | Isotonitazene/Protonitazene (0.01) |  |  |
|  | N-propionyl Norfentanyl (0.01) |  |  |
| 40 | Xylazine (1.50) |  |  |
|  | Fentanyl (1) |  |  |
|  | Quinine/Quinidine (0.85) |  |  |
|  | Diphenhydramine (0.75) |  |  |
|  | Caffeine (0.33) |  |  |
|  | 4-ANPP (0.22) |  |  |
|  | Tramadol (0.04) |  |  |
|  | Cocaine (0.04) |  |  |
|  | Acetaminophen (0.02) |  |  |
|  | Lidocaine (0.02) |  |  |
|  | Ethyl 4-ANPP (0.01) |  |  |
|  | Medetomidine (0.01) |  |  |
|  | Acetyl Fentanyl (0.01) |  |  |

| Supplemental Table 1 (Continued). | | | |
| --- | --- | --- | --- |
| 41 | Xylazine (1.88) | *Dermacoccus* | 452 |
|  | Fentanyl (1) | *Dermacoccus* | 451 |
|  | 4-ANPP (0.33) | *Yarrowia* | 453 |
|  | Diphenhydramine (0.14) |  |  |
|  | Quinine/Quinidine (0.06) |  |  |
|  | Norfentanyl (0.03) |  |  |
|  | 6MAM (0.02) |  |  |
| 42 | Xylazine (1.71) | *Dermacoccus* | 454 |
|  | Fentanyl (1) | *Dermacoccus* | 456 |
|  | 4-ANPP (0.38) | *Dermacoccus* | 455 |
|  | Diphenhydramine (0.15) |  |  |
|  | Quinine/Quinidine (0.08) |  |  |
|  | 6MAM (0.03) |  |  |
|  | Norfentanyl (0.02) |  |  |
|  | Cocaine (0.02) |  |  |
| 43 | Xylazine (2.13) | *Dermacoccus* | 458 |
|  | Fentanyl (1) | *Dermacoccus* | 457 |
|  | 4-ANPP (0.17) | *Candida* | 112 |
|  | Cocaine (0.10) |  |  |
|  | Diphenhydramine (0.05) |  |  |
|  | Quinine/Quinidine (0.02) |  |  |
|  | Norfentanyl (0.02) |  |  |
| 44 | Fentanyl (1) | *Dermacoccus* | 461 |
|  | 4-ANPP (0.18) | *Dermacoccus* | 460 |
|  | Cocaine (0.13) | *Aspergillus* | 464 |
|  |  | *Aspergillus* | 462 |
| 45 | Xylazine (1.61) | *Dermacoccus* | 468 |
|  | Fentanyl (1) |  |  |
|  | 4-ANPP (0.19) |  |  |
|  | Quinine/Quinidine (0.07) |  |  |
|  | Diphenhydramine (0.05) |  |  |
|  | 6MAM (0.02) |  |  |
|  | Norfentanyl (0.02) |  |  |
|  | Cocaine (0.02) |  |  |
|  | Acetyl Fentanyl (0.01) |  |  |
| 46 | Quinine/Quinidine (2.01) |  |  |
|  | Cocaine (1.81) |  |  |
|  | Diphenhydramine (1.28) |  |  |
|  | Fentanyl (1) |  |  |
|  | Xylazine (0.67) |  |  |
|  | 4-ANPP (0.59) |  |  |
|  | Caffeine (0.21) |  |  |

| Supplemental Table 1 (Continued). | | | |
| --- | --- | --- | --- |
| 47 | Fentanyl (1) | *Penicillium* | 489 |
|  | Xylazine (0.44) |  |  |
|  | Quinine/Quinidine (0.22) |  |  |
|  | 4-ANPP (0.19) |  |  |
|  | Cocaine (0.12) |  |  |
|  | Diphenhydramine (0.11) |  |  |
|  | Ethyl 4-ANPP (0.05) |  |  |
|  | Tramadol (0.04) |  |  |
|  | Caffeine (0.04) |  |  |
|  | Acetaminophen (0.01) |  |  |
| 48 | Fentanyl (1) | *Klebsiella* | 477 |
|  | Quinine/Quinidine (0.95) | *Klebsiella* | 479 |
|  | Diphenhydramine (0.90) | *Klebsiella* | 476 |
|  | 4-ANPP (0.27) |  |  |
|  | Caffeine (0.14) |  |  |
|  | Cocaine (0.13) |  |  |
|  | Xylazine (0.02) |  |  |
|  | 6MAM (0.02) |  |  |
| 49 | Fentanyl (1) | *Penicillium* | 475 |
|  | 4-ANPP (0.28) | *Penicillium* | 474 |
|  | Quetiapine (0.10) |  |  |
|  | Cocaine (0.09) |  |  |
|  | Diphenhydramine (0.05) |  |  |
|  | Quinine/Quinidine (0.02) |  |  |
|  | 6MAM (0.02) |  |  |
|  | Acetyl Fentanyl (0.01) |  |  |
|  | Heroin (0.01) |  |  |
|  | Morphine (trace) |  |  |
|  | Trazodone (trace) |  |  |
|  | Ethyl 4-ANPP (trace) |  |  |
|  | Norfentanyl (trace) |  |  |
|  | Xylazine (trace) |  |  |
| 50 | Quinine/Quinidine (2.15) | *Cladosporium* | 470 |
|  | Diphenhydramine (1.21) | *Cladosporium* | 472 |
|  | Fentanyl (1) |  |  |
|  | 4-ANPP (0.32) |  |  |
|  | Caffeine (0.19) |  |  |
|  | Cocaine (0.05) |  |  |

| Supplemental Table 2. Biofilm formation of *C. parapsilosis* isolates. | | | | | |
| --- | --- | --- | --- | --- | --- |
|  | n23 | n30 | n42 | n47 | Neg. control |
| 24°C | *1.923 | 2.211 | 2.275 | 2.114 | 0.042 |
| 37°C | 1.322 | 1.646 | 1.725 | 1.543 | 0.021 |
| * All readings were performed at an absorbance of 550 nm and numbers represent an average of three assays. | | | | | |

| Supplemental Table 3. Genes identified in loss-of-killing mutants. | | | | |
| --- | --- | --- | --- | --- |
| Mutant | Predicted Gene | JGI gene ID | Genome Coordinates | BGC |
| 1 | Adenylyltransferase | 8069812977 | 147,778-148,437 | No |
| 2 | Dehydrogenase | 8069813130 | 316,512-317,573 | No |
| 3 | Transcriptional regulator | 8069813539 | 769,447-770,991 | No |
| 4 | Argininosuccinate lyase | 8069813742 | 997,317-998,711 | No |
| 5 | AraC binding protein | 8069814891 | 2,213,238-2,213,975 | No |
| 6 | L-ornithine N5-oxygenase | 8069816793 | 4,240,910-4,242,244 | No |
| 7 | NRPS | 8069817298 | 4,758,672 - 4,771,625 | Yes |
| 8 | Sigma factor | 8069817299 | 4,772,025 - 4,772,555 | Yes |
