# Supplementary material for: Opportunistic pathogens and polycocktail drugs fuel dynamic public health threats during the opioid crisis
Source: PLoS One. 2025 Aug 12;20(8):e0326200. doi: 10.1371/journal.pone.0326200 (PMC12342250; doi:10.1371/journal.pone.0326200)
Supplement: S2 Table — (DOCX) [file pone.0326200.s002.docx]

| S2 Table. Biofilm formation of *C. parapsilosis* isolates. | | | | | |
| --- | --- | --- | --- | --- | --- |
|  | n23 | n30 | n42 | n47 | Neg. control |
| 24°C | *1.923 | 2.211 | 2.275 | 2.114 | 0.042 |
| 37°C | 1.322 | 1.646 | 1.725 | 1.543 | 0.021 |
| * All readings were performed at an absorbance of 550 nm and numbers represent an average of three assays. | | | | | |
