# Supplementary material for: Opportunistic pathogens and polycocktail drugs fuel dynamic public health threats during the opioid crisis
Source: PLoS One. 2025 Aug 12;20(8):e0326200. doi: 10.1371/journal.pone.0326200 (PMC12342250; doi:10.1371/journal.pone.0326200)
Supplement: S3 Table — (DOCX) [file pone.0326200.s003.docx]

| S3Table. Genes identified in loss-of-killing mutants. | | | | |
| --- | --- | --- | --- | --- |
| Mutant | Predicted Gene | JGI gene ID | Genome Coordinates | BGC |
| 1 | Adenylyltransferase | 8069812977 | 147,778-148,437 | No |
| 2 | Dehydrogenase | 8069813130 | 316,512-317,573 | No |
| 3 | Transcriptional regulator | 8069813539 | 769,447-770,991 | No |
| 4 | Argininosuccinate lyase | 8069813742 | 997,317-998,711 | No |
| 5 | AraC binding protein | 8069814891 | 2,213,238-2,213,975 | No |
| 6 | L-ornithine N5-oxygenase | 8069816793 | 4,240,910-4,242,244 | No |
| 7 | NRPS | 8069817298 | 4,758,672 - 4,771,625 | Yes |
| 8 | Sigma factor | 8069817299 | 4,772,025 - 4,772,555 | Yes |
