# Supplementary figures and images for: Opportunistic pathogens and polycocktail drugs fuel dynamic public health threats during the opioid crisis
Source: PLoS One. 2025 Aug 12;20(8):e0326200. doi: 10.1371/journal.pone.0326200 (PMC12342250; doi:10.1371/journal.pone.0326200)

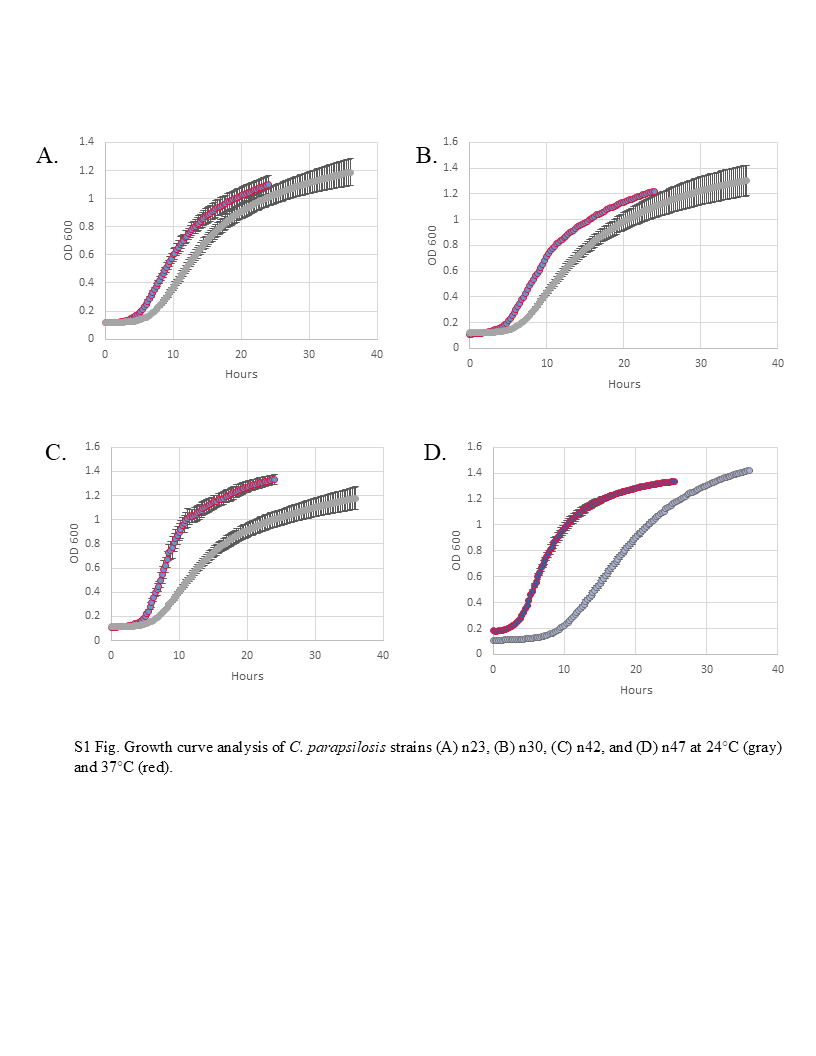

Supplement: S1 Fig — (TIF) [file pone.0326200.s004.tif]
